# Personality disorders and Juvenil Myoclonic Epilepsy

**DOI:** 10.1192/j.eurpsy.2022.1717

**Published:** 2022-09-01

**Authors:** I. Santos Carrasco, J. Gonçalves Cerejeira, M. Queipo De Llano De La Viuda, A. Gonzaga Ramírez, G. Guerra Valera, T. Jiménez Aparicio, C. De Andrés Lobo, C. Vallecillo Adame, M. Fernández Lozano, B. Rodríguez Rodríguez, N. Navarro Barriga, M.J. Mateos Sexmero, L. Gallardo Borge

**Affiliations:** 1 Clinical Hospital of Valladolid, Psychiatry, Valladolid, Spain; 2 Hospital Clínico Universitario de Valladolid, Psychiatry, Valladolid, Spain; 3 Hospital Clínico Universitario, Psiquiatría, Valladolid, Spain; 4 Hospital Clínico Universitario de Valladolid, Psiquiatría, VALLADOLID, Spain

**Keywords:** juvenile myoclonic epilepsy, therapeutic adherence, Personality disorders

## Abstract

**Introduction:**

There is a high comorbidity between psychiatric disorders and juvenile myoclonic epilepsy (JME), observed in up to 58% of these patients; specifically, mood disorders, anxiety and personality disorders (PD). In some patients with PD there are nonspecific alterations in the EEG, which nevertheless sometimes involve pathology. The presence of personality disorders along with JME has been repeatedly described. Previous studies have emphasized the difficulties in treating patients with JME, which have been attributed to some specific psychiatric, psychological and psychosocial characteristics.

**Objectives:**

Describing distinctive personality traits in JME

**Methods:**

Review of scientific literature based on a relevant clinical case.

**Results:**

19-year-old woman, single. Psychiatric history since she was 12 due to anxiety-depressive symptoms, after being diagnosed with JME. 4 admissions in Psychiatry, with a variety of diagnoses: eating disorder, attention deficit hyperactivity disorder and borderline personality disorder. The evolution of both disorders has been parallel, presenting epileptic seizures due to irregular therapeutic adherence together with pseudo-seizures, which made difficult their differential diagnosis. In addition, he has had frequent visits to the emergency room for suicide attempts and impulsive behaviors.

**Conclusions:**

In 1957, for the first time, distinctive personality traits were described in patients with JME: lack of control and perseverance, emotional instability, variable self-concept and reactive mood, which have been confirmed in subsequent studies. It is believed as epilepsy progresses, patients tend to develop symptoms of depression, anxiety, social problems, and attention deficit. Therefore, these patients have difficulty in following medical recommendations, especially precautions regarding precipitating factors for seizures.

**Disclosure:**

No significant relationships.

